# Diabetes, Albuminuria and the Kidney—Brain Axis

**DOI:** 10.3390/jcm10112364

**Published:** 2021-05-27

**Authors:** Diana Maria Ariton, Joan Jiménez-Balado, Olga Maisterra, Francesc Pujadas, María José Soler, Pilar Delgado

**Affiliations:** 1 Neurology Department, Hospital Vall d’Hebron, Universitat Autònoma de Barcelona, 08035 Barcelona, Spain; dm.ariton25@gmail.com (D.M.A.); joan.balado@gmail.com (J.J.-B.); omaisterra@vhebron.net (O.M.); fpujadas@vhebron.net (F.P.); 2 Leonard Davis School of Gerontology, University of Southern California, Los Angeles, CA 90089, USA; 3 Nephrology Department, Hospital Vall d’Hebron, Universitat Autònoma de Barcelona, 08035 Barcelona, Spain; mjsoler01@gmail.com

**Keywords:** dementia, cognitive decline, albuminuria, chronic kidney disease, diabetes

## Abstract

Cognitive decline and kidney disease are significant public health problems that share similar characteristics and risk factors. The pathophysiology of the kidney–brain axis is not completely understood, and studies analysing the relationship between the biomarkers of kidney damage and cognitive impairment show different results. This article focuses on the epidemiological and clinical aspects concerning the association of albuminuria, a marker for endothelial dysfunction and microvascular disease, and cognitive impairment in patients with chronic kidney disease, diabetic kidney disease and end-stage kidney disease. Most studies show a positive relationship between albuminuria and cognitive impairment in all groups, but evidence in type 2 diabetes (T2D) patients is limited. We briefly discuss the mechanisms underlying these associations, such as damage to the microvascular circulation, leading to hypoperfusion and blood pressure fluctuations, as well as increased inflammation and oxidative stress, both in the brain and in the kidneys. Further clinical and epidemiological studies developed to understand the interplay between the kidneys and brain diseases will hopefully lead to a reduction in cognitive impairment in these patients.

## 1. Introduction

According to the World Health Organization, dementia is a syndrome in which there is deterioration in cognitive functions beyond what might be expected from normal ageing, and it is occasionally preceded by deterioration in emotional control, social behaviour or motivation. There were over 50 million people worldwide living with dementia in 2020, and this number is expected to double every 20 years, reaching 152 million in 2050, with an estimated annual global cost greater than US$ 1 trillion [1]. As human life expectancy increases, dementia and cognitive decline are becoming more prevalent in older adults, leading to a reduced quality of life, loss of independence, heavier caregiver burden and premature mortality [2]. Therefore, the early identification of patients at higher risk is essential to achieve better surveillance and earlier diagnosis to conduct effective preventive and treatment strategies for dementia [3].

Among these high-risk patients, those with diabetes and/or kidney disease might be particularly interesting, given that both are clinical conditions with a higher prevalence of cognitive disorders than what is present in the general population. In chronic kidney disease (CKD), the prevalence and degree of cognitive decline increase with advanced CKD stages [4,5,6].

Chronic kidney disease is characterized by a urinary albumin:creatinine ratio (UACR) ≥ 30 mg/g and/orestimated glomerular filtration rate (eGFR) < 60 mL/min per 1.73 m^2^. CKD is an increasingly recognized public health problem, affecting more than 13% of the worldwide population with higher rates in developed areas [7].

Type 2 diabetes (T2D), which accounts for 90% of the global burden of diabetes, is the most frequent cause of kidney complications [8]. Diabetic kidney disease (DKD) is a clinical diagnosis based upon the presence of albuminuria, a decreased estimated glomerular filtration rate (eGFR) or both in diabetes. People with diabetes have a nearly two-fold higher risk of CKD than those without diabetes. In addition, in many countries, including the United States, diabetes is responsible for over 40% of new cases of end-stage renal disease (ESKD). The UK Prospective Diabetes study reported that 38% of people with newly diagnosed type 2 diabetes developed albuminuria during a median follow-up of 15 years and that 29% developed renal impairment [9].

Albuminuria, which is a type of proteinuria wherein the protein albumin is abnormally present in the urine (usually, only trace amounts of albumin can be found in urine, as it is a major plasma protein that normally circulates in the blood), is considered a marker for microvascular and kidney damage, as well as an early indicator of diabetic kidney disease. According to the amount of urine protein content, albuminuria is divided into the following three categories according to the albumin–creatinine ratio (ACR) or the 24-h albumin excretion rate (AER): A1 or normoalbuminuria (<30 mg/g or <30 mg/24 h), A2 or microalbuminuria (30–300 mg/g or 30–300 mg/24 h) and A3 or macroalbuminuria (>300 mg/g or >300 mg/24 h) [10].

Over the past few years, despite a wide variability in the methodologies and outcome assessments, several studies have shown associations between albuminuria and the risk of developing cognitive impairment and dementia, as well as a correlation with imaging markers for cognitive decline. Moreover, a recent meta-analysis provided the same conclusions [11,12,13,14,15,16,17,18,19,20,21] based on the results of population-based studies. Less is known about T2D specifically; however, because T2D patients suffer from both an increased risk of cognitive impairment and kidney disease [22,23,24,25,26,27,28], elucidating the mechanisms of disease and exploring the interplay between the brain and kidney might potentially help to prevent cognitive impairment both in general and T2D populations.

The present study involved a literature review concerning epidemiological and clinical studies assessing the relationship between albuminuria and cognitive decline or dementia, first in general populations and, second, in T2D. We provided a summary of the main mechanisms underlying these associations and examined the different treatment strategies aimed at reducing cognitive impairment in diabetes.

## 2. Materials and Methods

A literature search was conducted using PubMed up to January 25, 2021. The search strategy included terms related to predictors (‘’albuminuria’’, ‘’proteinuria’’, urine albumin-to-creatinine ratio’’ or ‘’UACR’’ or “ACR”) and terms for the outcomes (‘’dementia’’, ‘’cognitive impairment’’ or Alzheimer’’) without language or type of article restrictions. From all articles initially identified that fulfilled these criteria, we focused on the studies with a clear outcome on incident dementia and cognitive function, preferably with prospective and longitudinal designs, as well as with detailed references to either screening or more comprehensive standardized methods to assess cognition.

## 3. Results

### 3.1. Studies on Albuminuria, Cognitive Impairment and Dementia in the General Population

The first time albuminuria was linked to brain functions was in 1989, when the ‘’Steno hypothesis” [29] was published. This theory was derived from type 1 diabetic patients with albuminuria, and at that time, albuminuria was proposed as a biomarker of generalized endothelial dysfunction related to an increase in vascular events, in addition to being a local renal damage marker. Since then, multiple studies have been published involving diabetic and nondiabetic populations, showing an independent correlation between albuminuria and cognitive impairment.

Two recent meta-analyses on renal dysfunction, cognition and the risk of dementia showed an increased risk of up to 35% for cognitive decline and dementia related to albuminuria, but it remained unclear whether the relationship is causal or due to common pathophysiological mechanisms [21,30].

Following these meta-analyses, several studies have contributed additional new information to overcome the limitations noted in previous studies. Nevertheless, it remains uncertain whether the effect of albuminuria is independent of shared risk factors (i.e., cardiovascular risk factors, obesity and lifestyle habits), because most studies did not adjust for these confounders. In a 12-year follow-up study conducted in an Australian population [31], Sacre et al. confirmed that the effect of albuminuria on the processing speed is only derived from cardiovascular risk factors (CVRFs), whereas the effect on memory is driven by albuminuria itself. Another longitudinal study in a Finnish population also observed similar results with UACR, even at levels below the definition of microalbuminuria. In this case, the researchers reported that the associations are attenuated with adjustment by CVRF, but they are still present [32]. Therefore, there might be a differential effect of albuminuria and CVRF in specific cognitive domains. 

The Atherosclerosis Risk in Communities (ARIC) study investigated whether the effect of albuminuria on cognitive decline exists across the lifespan and whether its magnitude is stronger in midlife than in elderly individuals (as happens with other vascular risk factors, such as hypertension, whose effect is stronger at midlife). In the ARIC study, a consistent association of microalbuminuria and dementia was reported in both midlife (HR 1.15) and late life (HR 1.27); although the effect was stronger in late life, there were no differences between the associations at midlife and late life. In addition, the risk was higher when albuminuria was combined with a decrease in renal function (eGFR). Beyond microalbuminuria, a significant association was found between diabetes in midlife and dementia [11]. These results were similar to those obtained by other researchers, such as Joosten et al., who demonstrated a significant association between albuminuria and cognitive impairment in patients under 49 years of age, but the association was not statistically significant above this age [33].

The results from the SCOPE study [34], in contrast, showed no effect of kidney disease on cognition in late life. However, this study was conducted in a population older than 75 years old, in which the prevalence of end-stage CKD might be low due to a survival bias in which individuals with advanced CKD and possibly cognitive impairment already died and, therefore, were not included.

Finally, some studies have investigated the association of albuminuria with different dementia subtypes. Alzheimer’s disease (AD) is the most frequent subtype, corresponding to 60–80% of all cases, followed by vascular dementia (VaD), dementia with Lewy bodies (DLB) and other neurodegenerative dementias, including a mixture of the first two previous ones [35]. After a 16-year follow-up, Gabin et al. [14] showed a positive association between the microalbuminuria and different subtypes of dementia, such as Alzheimer’s disease, vascular dementia, a mixture of AD/VaD and other dementias, including DLB and FTD. These researchers found a positive association between albuminuria and AD/VaD, with stronger associations for VaD (HR = 3.97) upon the subgroup analysis. These results were consistent with the previous literature based on the hypothesis of a shared microvascular pathology in the kidneys and the brain. In line with these results, the Hisayama study [16] was conducted in individuals from Japan who were older than 60, and the primary outcome was the global incidence of dementia and dementia subtype. Apart from the UACR measurement obtained at the baseline and over the follow-up, this study included a neuropathological assessment in most participants (*n* = 215) and neuroimaging (*n* = 319). Therefore, the underlying aetiology of dementia was confirmed in most cases. To our knowledge, this is one of the few studies reporting the association of albuminuria and different dementia aetiologies with pathological confirmation. The cumulative incidence of all-cause dementia for both AD and/or VaD was significantly increased with higher UACR levels. Interestingly, a stratified analysis showed a significant association between UACR and the development of VaD, especially when a prior stroke event was confirmed. These results are in line with the importance of albuminuria as a surrogate marker of endothelial dysfunction and atherosclerotic vascular disease.

Nevertheless, few studies [11,31] have determined whether albuminuria is persistent or disappears during follow-up as a result of treatments or changes in lifestyle [36]. Therefore, future longitudinal studies with multiple determinations of albuminuria are needed to clarify this point.

Additionally, there is wide variability across the studies in the cognitive assessments (Table 1), ranging from studies using simple screening tests to studies using neuropsychological batteries exploring several cognitive domains. In this regard, some studies have found a close relation between albuminuria and memory [11,14,16,25,26,31]. However, others have not found a close relation but, instead, have found associations with the processing speed or executive functions. The diagnostic criteria for dementia and cognitive impairment are also variable. Standardized neuropsychological evaluations and uniform diagnostic criteria for cognitive impairment and dementia may lead to more easily reproducible results between studies with similar characteristics in the future.

### 3.2. Albuminuria, Mild CKD, Cognitive Impairment and Dementia in T2D

The relationship between diabetic kidney disease (DKD) and cognitive impairment may involve multiple factors, including metabolic, vascular and sociodemographic factors.Epidemiological studies focusing specifically on T2D patients have been conducted in the past. After a review of studies that have assessed the risk for impaired cognitive performance in the presence of DKD, Ghoshal et al. concluded that, in patients with DKD, the presence of higher albuminuria, lower eGFR and markers of systemic inflammation contribute to the relationship between nephropathy and cognitive impairment [23]. Sincemost studies have been conducted on European and non-African American cohorts, and previous studies have suggested a differential effect of ethnicity (with an accelerated cognitive decline in African Americans compared to other cohorts), Freedman et al. [25] conducted a cross-sectional analysis in African American T2D individuals with normal or mildly impaired kidney function and low albuminuria levels, providing similar results to European cohorts after adjustment for vascular risk factors and diabetes duration.

A cross-sectional study conducted in a Filipino population with an average 12-year T2D duration observed a higher rate of mild cognitive impairment (45%) than previous studies among Korean and Polish populations, which described lower prevalences (31.5% and 32.7%, respectively) [26]. Furthermore, patients in the Filipino study reported poor results in language and recall, in contrast with previous studies that observed a decline in executive functions [37,38,39].

For middle-aged T2D patients [22], evidence has shown an independent and negative correlation between albuminuria and cognitive function, even with a short development time for the disease (mean duration of <5 years), which may be due to a possible contribution of albuminuria at the beginning of cognitive deterioration, which, added to the rest of the classically associated factors, would end up producing the cognitive impairment observed in older patients who have a longer progression time for diabetes mellitus.

A review of the literature indicated that there are few studies exploring the effect of albuminuria and kidney function on cognitive impairment in type 2 diabetic patients compared to general population studies. Even in clinical trials for T2D that have evaluated cognition as a secondary outcome, the contribution or interactions with albuminuria or kidney function have not been investigated in depth. Given the clinical implication of the observed findings and frequent coexistence of diabetes and kidney dysfunction, future studies will need to address this fact to develop and implement future treatment strategies.

The principal highlights of the reviewed studies are summarized in Table 1.

### 3.3. End-Stage Kidney Disease, Dialysis and Cognitive Impairment

The estimated prevalence of cognitive impairment in this group ranges between 30% and 60%. We reviewed the latest publications on kidney disease and cognitive performance in patients with diabetes, especially diabetic patients with ESKD. Ghoshal and collaborators observed a faster decline in cognitive function in patients with known cardiovascular disease or DKD. Vascular mechanisms have an important role in cognitive decline in these individuals, because patients with ESKD, diabetes and ongoing dialysis have a particularly high risk of stroke, especially intradialytic ischaemic and haemorrhagic stroke. Moreover, post-stroke patients with ESKD have poorer functional outcomes, and patients with DKD have nearly four times the risk of cognitive decline when compared to patients without kidney dysfunction or T2D [23,40].

### 3.4. Pathophysiology and Mechanisms

#### 3.4.1. Similarities between Brain and Kidney

As albuminuria is a risk factor for cognitive impairment, we further reviewed several hypotheses that might mechanistically explain this link. First, from a systemic point of view, the brain and kidney present several similarities in their vascular system. Both are considered low-resistance end-organs exposed to a high volume of blood flow [41]. These common haemodynamic characteristics make the brain and kidney vascular beds especially vulnerable to fluctuations in blood pressure [42]. Moreover, brain arterioles arising from perforating arteries are morphologically similar to kidney juxtamedullary arterioles, and both are responsible for maintaining a strong vascular tone to provide a good pressure gradient from parent vessels to capillaries [43,44]. Therefore, both organs are highly exposed to risk factors, such as hypertension and diabetes, which lead to damaged large and small vessels. The loss of vascular autoregulation and poorer vascular tone in both the brain and kidneys might result in an elevated vulnerability to hypoperfusion and blood pressure fluctuations [41]. In contrast, hyperglycaemia triggered by diabetes causes significant endothelial damage mediated by increased inflammatory processes, oxidative stress and sorbitol production [45]. However, this relationship might be bidirectional, as correct endothelial permeability is required for insulin transport, suggesting that endothelial damage may worsen glucose metabolism in diabetic individuals [46,47].

Hypertension and diabetes, as well as other factors, either independently or in combination, result in reduced filtration in the kidneys, albuminuria and cerebral small vessel disease (cSVD) in the brain [48]. The term cSVD refers to all the pathological changes that affect brain arterioles, venules and capillaries. The principal manifestation of cSVD is the appearance of subclinical cerebrovascular lesions on the brain parenchyma, including WMHs, lacunar infarcts, cerebral microbleeds, enlarged perivascular spaces and cortical atrophy [49]. In both organs, the effects of hypertension and diabetes in the small vessels should be considered as chronic conditions evolving within the clinical spectrum [50]. In the brain, the accumulation of WMHs and other radiological markers of cSVD is known to increase the risk of clinical stroke and cognitive impairment [51,52], while, in the kidneys, there is a continuous decline in the eGFR over the course of years. However, the loss of kidney function is not a linear process; for instance, microvascular disease is considered to produce a hyperfiltration state at the initial states of the disease, which, in turn, encompasses the appearance of microalbuminuria and increased glomerular capillary pressure [53]. Of note, hyperfiltration is especially common in prediabetic and type II diabetic individuals [54]. In addition, patients with CKD might experience a significant long-term variation in eGFR, and this variability has been associated with an increased risk of dementia [55]. Finally, during the later stages of CKD, there is also hyperfiltration at the single-nephron level to compensate for the loss of nephrons, which ultimately results in an accelerated decline in renal function [53].

Hence, the brain and kidney exhibit parallel impairments due to shared vulnerabilities, and this is confirmed by the results from our group, in which we showed that the progression of periventricular WMH over 4 years wasrelated to the changes in albuminuria, suggesting that albuminuria and cerebral small vessel disease progress in parallel [56]. Nonetheless, beyond this parallel course, it is difficult to demonstrate a causal link between cSVD and CKD, as most results originate from observational studies in which the conclusions are confounded by reversed causation and concomitancy. To overcome these limitations, Marini and collaborators (2020) performed a Mendelian randomization study and found that increased UACR and reduced eGFR are causally involved in large artery stroke, emphasizing that both large and small artery strokes share common genetic mechanisms with kidney disease [56]. To the best of our knowledge, no group has investigated whether UACR or eGFR is causally associated with subclinical cSVD or cognitive impairment via genetic studies, and future reports will be of great interest in this regard.

#### 3.4.2. Albuminuria as a Marker of Endothelial Dysfunction

From a biological point of view, however, several hypotheses may link albuminuria to cSVD pathogenesis and, thus, to cognitive impairment onset. UACR is a marker of endothelial dysfunction, reflecting systemic damage in the microcirculation. In this sense, a study from the Maastricht cohort evaluated several markers of microvascular disease (including albuminuria but, also, others involving different organs, such as the retina and brain) and found an association with memory and processing speeds [57]. Furthermore, one meta-analysis, pooling results from 27 studies (mainly cross-sectional), confirmed the association between microalbuminuria and cSVD [21]. These articles provided compelling evidence of UACR as a surrogate marker of microvascular disease, which leads to increased cerebrovascular burden and cognitive decline.

Importantly, damage to the endothelium is one of the key mechanisms triggering the blood–brain barrier (BBB) breakdown, one of the principal agents in the pathogenesis of cSVD [58]. Endothelial cells present tight junctions preventing the entry of hydrophilic substances into the brain [59], and together with neurons, astrocytes, microglia and pericytes, they form the neurovascular unit [60,61]. Hence, the maintenance of neurovascular coupling is crucial to maintaining the correct brain functions and a balanced transfer of substances within the BBB [62].

Albuminuria represents a marker of microvascular disease preceding the loss of kidney function, accumulation of cSVD lesions and stroke [36,63,64,65]. After BBB breakdown occurs, several mechanisms may explain the connection between UACR and cognitive impairment, including an exacerbated inflammatory response. Indeed, inflammation plays a crucial role in vascular and neurodegenerative diseases via processes such as atherogenesis and platelet aggregation [66]. Interestingly, several blood inflammatory biomarkers have been associated with both cSVD and CKD, such as C-reactive protein (CRP), interleukin-1 (IL-1), IL-6 and tumour necrosis factor-α (TNF-α). Cross-sectional studies have found that TNF-α, IL-1, IL-6 and CRP are associated with WMH, silent brain infarcts, lower eGFR levels and a higher UACR [67,68,69]. Hence, there is an overlap between inflammatory markers in the brain and kidneys, suggesting a parallel course of inflammation in both organs.

#### 3.4.3. CKD and Neurodegenerative Diseases

As CKD worsens over time due to the cumulative effects of endothelial dysfunction and inflammation, the loss of kidney function may increase the likelihood of neurodegenerative diseases, such as AD. AD is characterized at the extracellular level by the aggregation of amyloid-β (Aβ) peptide in Aβ plaques and at the intracellular level by the hyperphosphorylation of tau in neurofibrillary tangles [70]. Aβ and tau act together over time, leading to neuronal dysfunction and death, which is observed as cortico–subcortical atrophy, especially in medial temporal regions, such as the hippocampus [71,72]. Interestingly, Aβ is systemically cleared by the liver and kidneys [58,73]. Importantly, Aβ clearance from the brain to blood within the BBB is insufficient, and its efficient systemic removal is necessary to maintain the Aβ levels [74,75]. Several studies have shown that reduced eGFR levels are associated with increased plasma Aβ in patients with CKD [76]. Individuals with mild CKD present higher serum Aβ levels than healthy older adults, and interestingly, haemodialysis might help to reduce these increased levels [77]. However, more research is needed to understand the effect of UACR and kidney dysfunction in AD biomarkers, especially in the general population.

Several structural MRI studies have found markers of CKD associated with brain atrophy in AD signature regions, providing further evidence of the link between AD-related pathology and kidney function. Kawamura and colleagues (2016) found that both UACR and eGFR predict the loss of hippocampal volume after a 3-year follow-up in diabetic patients, and this association remains after correcting for potential confounders, such as cSVD burden. Another cross-sectional study has found that individuals with CKD present a lower hippocampal volume and cortical thickness than matched controls [78]. Finally, Chen et al. (2020) confirmed that cortical thinning mediates the relationship between CKD and cognitive decline [79].

**Figure 1:**
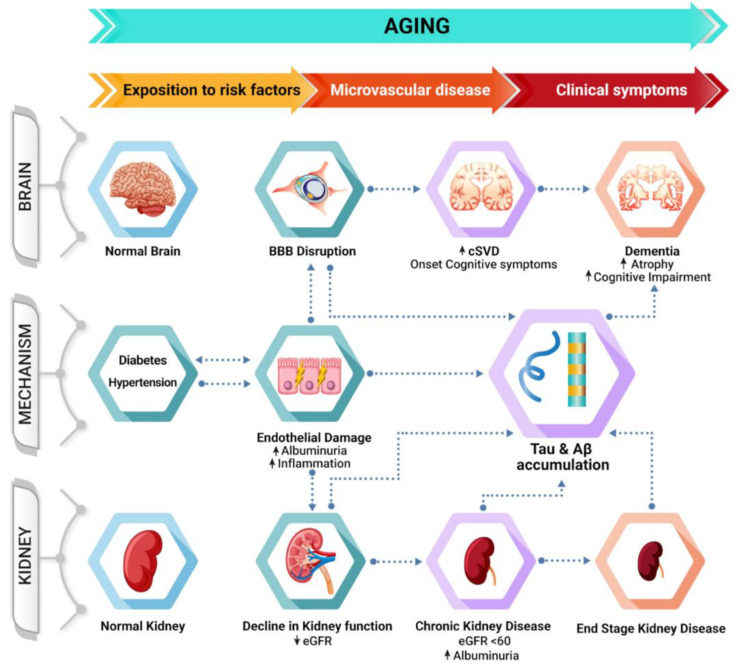
Schematic representation of the potential mechanisms linking albuminuria and cognitive impairment. Chronic exposure to risk factors during mid-life triggers endothelial damage, manifesting as increased albuminuria and leading to BBB disruption and decreased eGFR, which ultimately result in increased cerebrovascular burden and CKD. Additionally, decreased kidney function might compromise the systemic clearance of Aβ, increasing the likelihood of dementia during the later stages of the disease.Key: AD, Alzheimer’s disease; BBB, blood–brain barrier; CKD, chronic kidney disease; cSVD, cerebral small vessel disease; and eGFR, estimated glomerular filtration rate.

As most results come from observational studies, the exact mechanism linking kidney function, albuminuria and brain health is unknown. However, this relationship likely includes multicausal factors, with the initial trigger being exposure to vascular risk factors, such as diabetes and hypertension, during mid-life (Figure 1). Several studies have suggested that this sustained exposure yields endothelial damage, which will impair the brain and kidney via several mediating factors (i.e., inflammation and BBB disruption), and it manifests as increased albuminuria. After CKD onset, however, it is unknown how reduced kidney filtration compromises Aβ clearance in the general population. Moreover, it is also unknown whether reduced eGFR impacts Tau aggregation. Sincedisease-modifying treatments in AD are not yet available, it is of great interest to explore new methods to reduce AD-related pathology to reduce the incidence of dementia. Hence, we will further discuss the treatment strategies in patients with CKD and their effects on both kidney function and cognition.

### 3.5. Treatment Strategies

Treatment strategies for patients with mild-to-moderate chronic kidney disease have been recently reviewed by Drew and collaborators [80]. In general, control of the classic vascular risk factors is advised, because they improve cognitive functioning in the general population. Additionally, there are two specific interventions mentioned by these authors that may decrease the rate of cognitive decline. The first target to consider is the reduction of albuminuria with angiotensin-convertingenzyme (ACE) inhibitors or angiotensin-receptor blockers (ARBs) based on evidence from a post hoc analysis of the ONTARGET/TRANSCEND trials, 25% of the participants in which had CKD [81]. Additionally, given the associations between the renin angiotensin system and Alzheimer’s disease pathogenesis, several trials are currently testing a number of renin angiotensin system-acting drugs in patients at risk of Alzheimer’s disease [82].

The SPRINT MIND trial showed that intensive blood pressure reduction (compared to the standard BP-lowering targets) reduced the incidence of mild cognitive impairment (but not dementia), and importantly, this effect was not modified by the presence of CKD [83].

With regard to diabetic patients, the pathophysiology of cognitive decline in diabetes might be influenced by many interrelated factors. Hyperglycaemia may play a role, but in elderly individuals with diabetes, both hypoglycaemia and hyperglycaemia may lead to detrimental effects on cognition. In this regard, a systematic review and meta-analysis performed in 2017 explored the effect of different interventions (either lifestyle modifications and/or standard versus intensive blood glucose lowering) to achieve normoglycaemia and showed that none of the interventions achieved a significant reduction in the risk of cognitive impairment [84].

The Look AHEAD (Action for Health in Diabetes) study for type 2 diabetes, an intensive lifestyle intervention for overweight or obese participants comprising weight loss and exercise, found a significant improvement in kidney outcomes (measured by albuminuria and estimated GFR) and brain white matter hyperintensities, but no effect was noted on cognition [85].

In addition to glucose control in diabetes, evidence from experimental studies has proposed pleiotropic effects (i.e., not directly related to their glucose-lowering effect) for some antidiabetic drugs.There are conflicting results for the use of insulin and metformin, with independent studies showing the risks and benefits [86,87,88,89].

Experimental evidence also supports the use of dipeptidyl peptidase-4 inhibitors or gliptins as potential drugs to prevent cognitive impairment through the preservation of Glucagon-like peptide 1 (GLP-1) function and increasing neurogenesis, among many other mechanisms [90]. However, the translation into clinical trials has failed, as has been shown recently [91]. In the CAROLINA-COGNITION study [91], 3163 diabetic participants (40 to 85 years old; HbA1c range of 48–69 mmol/mol (6.5–8.5%) and who received standard care, excluding insulin therapy) were randomized to linagliptin versus glimepiride and followed-up for a mean duration of 6.1 years. The study assessed the effect of linagliptin and glimepiride on accelerated cognitive decline in participants with a baseline MMSE score ≥24. Approximately 28% of the participants experienced an accelerated cognitive decline at the follow-up in both treatment groups. The treatment was found to be safe, but it did not positively affect the cognitive functions, as was hypothesized in view of experimental studies. Of note, no differences were observed considering albuminuria as prespecified subgroups.

For pioglitazone, a synthetic ligand of peroxisome proliferator-activated receptors, and liraglutide, a GLP-1 receptor agonist, experimental and observational studies in humans showed neuroprotective effects by diminishing the cognitive decline, but more research is needed in clinical trials [92,93].

Finally, sodium–glucose cotransporter (SGLT2) inhibitors have shown a beneficial effect on the cognitive functions in experimental studies as well. This effect may be, at least in part, mediated by an increase in the neurotrophic factors and acetylcholinesterase-inhibiting activity, as well as a reduction in the amyloid burden and tau pathology in the cerebral cortex, and this effect may also be mediated by improvements in cardiovascular injury [94].

In summary, future trials in diabetes may benefit from exploring albuminuria and kidney function as mediators and/or secondary outcomes to better understand and study the brain–kidney axis.

## Figures and Tables

**Table 1:** Contributions of cognitive impairment and dementia in the general population and the type 2 diabetic population.

Authors	Sample; Mean Age, year; Follow-Up, year	Main Outcome	Cognitive Function and Cognitive Impairment Diagnosis	New Inputs
Sacre et al. 2019 [31]	Population-based AusDiab Study (*n* = 4128); ≥25; 12 years	Cognitive function	CVLT, SDMT	Albuminuria is related to memory impairment, while the effect of albuminuria on processing speed was only derived from cardiovascular risk factors
		Cognitive impairment when >60 years	MMSE when >60 years	
Ekblad et al. 2018 [32]	Finnish health community (*n* = 3687); ≥30; 11 years	Cognitive function	Verbal fluency, word-list learning, word-list delayed recall, simple and visual choice reaction time tests	Levels below microalbuminuria cut-off relate to cognitive function
Scheppach et al. 2020 [11]	Population from the ARIC study (*n* = 4626); 45–64; 5 to 20 years	Dementia	Neuropsychological battery, informant interviews, TICSm, surveillance based on prior discharge hospitalization ICD-9 or death certificate code for dementia	Consistent association of microalbuminuria and dementia in both midlife and late life
Gabin et al. 2019 [14]	Data from the HUNT 2 Study and the Health and Memory Study (*n* = 48,508); ≥20; 16 years	AD, VaD, AD/VaD and other	ICD-10 criteria based on clinical examination, patient, caregiver history and diagnostic imaging	Positive association between microalbuminuria and subtypes of dementia (AD, VaD, a mixture of AD/VaD and another dementia, including DLB and FTD
Takae K et al. 2018 [16]	Community-dwelling Japanese population (*n* = 1562); ≥60, 10 years	All-cause dementia and its subtypes (AD and VaD)	Clinical diagnosis: Guidelines of the Diagnostic and Statistical Manual of Mental Disorders (3rd Ed), NINCDS, ADRDA and NINDS-AIRENNeuroimaging: MRI and CTAutopsy	UACR is associated with increased cumulative incidence of all-cause dementia (pathologically confirmed), especially VaD
Tap L et al. 2020 [34]	SCOPE study cohort (*n* = 2252); ≥75; 2 years	Cognitive disfunction and cognitive impairment	MMSE	No relation between kidney disease and late life cognitionPossible lack of end stage kidney disease population and survival bias
Blanquisco et al. 2017 [26]	General population from the General Medicine and Diabetes Clinics of the Philippine General Hospital (*n* = 133); ≥60 years; cross-sectional	Mild Cognitive Impairment	MoCA-P	Positive association between albuminuria and mild cognitive impairment with poor results in language and recall in patients with T2D
Barzilay JI et al., 2020 [22]	GRADE study (*n* = 4998); ≥20; 4 to 7 years	Cognitive function	Spanish English Verbal Learning Test, Letter and Animal fluency tests, Digit Symbol Substitution Test	Middle-aged adults with kidney disease and T2D have cognitive decline even in short duration diabetes
Freedman et al., 2017 [25]	Cohort from the AA-DHS MIND (*n* = 512) and ACCORD (*n* = 484) cross-sectional	Cognitive performance	MMSE, Digit Symbol Coding Stroop Test and Rey Auditory Verbal Learning Test.MRI	Possible differentiation based on upon ancestry on albuminuria and cognition results in patients with T2D

CVLT: California Verbal Learning Test; SDMT: Symbol Digit Modalities Test; MMSE: Mini-Mental State Examination; TICSm: Telephone Interviews for Cognitive Status-Modified; ICD-9: International Classification of Diseases, Ninth Revision; ICD-10: International Classification of Diseases, Tenth Revision; MoCA-T: Taiwanese Version of the Montreal Cognitive Assessment; WMS-III: Taiwanese Version of the Wechsler Memory Scale-Third Edition; NINDS: National Institute of Neurological and Communicative Disorders and Stroke; ADRDA: Alzheimer’s Disease and Related Disorders Association, NINDS-AIREN: National Institute of Neurological Disorders and Stroke-Association Internationale pour la Recherche et l’Enseignementen Neurosciences; MoCA-P: The Montreal Cognitive Assessment-Philippines Tool; MRI: Magnetic Resonance Imaging; CT: Computed Tomography.
